# Transformation of Lipid Vesicles into Micelles by Adding Nonionic Surfactants: Elucidating the Structural Pathway and the Intermediate Structures

**DOI:** 10.1021/acs.jpcb.1c09685

**Published:** 2022-03-14

**Authors:** Igor Kevin Mkam Tsengam, Marzhana Omarova, Elizabeth G. Kelley, Alon McCormick, Geoffrey D. Bothun, Srinivasa R. Raghavan, Vijay T. John

**Affiliations:** † Department of Chemical and Biomolecular Engineering, Tulane University, 300 Lindy Boggs Building, New Orleans, Louisiana 70118, United States; ‡ Center for Neutron Research, National Institute of Standards and Technology, Gaithersburg, Maryland 20899, United States; § Department of Chemical Engineering and Material Science, University of Minnesota, 421 Washington Avenue SE, Minneapolis, Minnesota 55455, United States; ∥ Department of Chemical Engineering, University of Rhode Island, 51 Lower College Road; Kingston, Rhode Island 02881, United States; ⊥ Department of Chemical and Biomolecular Engineering, University of Maryland, College Park, Maryland 20742, United States

## Abstract

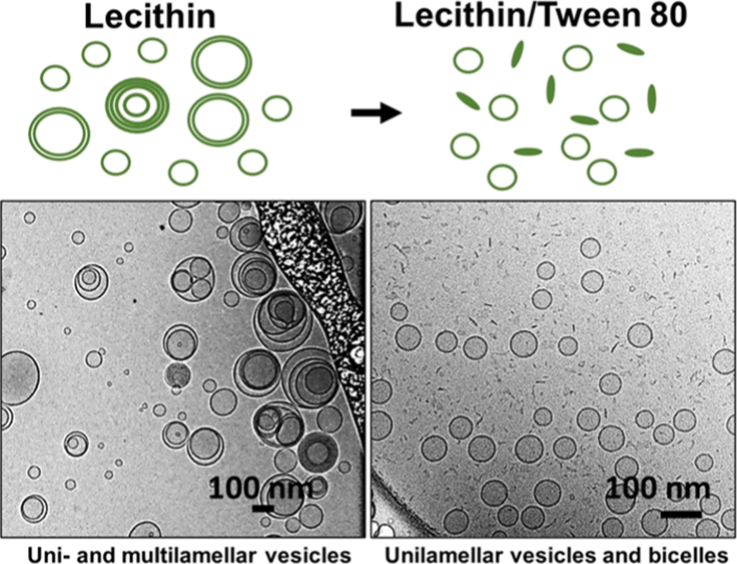

The phospholipid lecithin (L) and the nonionic surfactant Tween 80 (T) are used together in various contexts, including in drug delivery and oil spill remediation. There is hence a need to elucidate the nanostructures in LT mixtures, which is the focus of this paper. We study these mixtures using cryogenic transmission electron microscopy (cryo-TEM), coupled with dynamic light scattering and small-angle neutron scattering. As the concentration of Tween 80 is increased, the vesicles formed by lecithin are transformed into spherical micelles. We identify bicelles (i.e., disc-like micelles) as well as cylindrical micelles as the key stable nanostructures formed at intermediate L/T ratios. The bicelles have diameters ∼13–26 nm, and the bicelle size decreases as the Tween 80 content increases. We propose that the lecithin lipids form the body of the discs, while the Tween 80 surfactants occupy the rims. This hypothesis is consistent with geometric arguments because lecithin is double-tailed and favors minimal curvature, whereas the single-tailed Tween 80 molecules prefer curved interfaces. In the case of cylindrical micelles, cryo-TEM reveals that the micelles are short (length < 22 nm) and flexible. We are able to directly visualize the microstructure of the aggregates formed by lecithin–Tween 80 mixtures, thereby enhancing the understanding of morphological changes in the lecithin–Tween 80 system.

## Introduction

The aqueous self-assembly of amphiphile mixtures is a subject of significant research interest.^1^ The self-assembly of single amphiphiles in water is driven by a balance between the hydrophobic interactions of the tails and the geometrical packing constraints of the head groups.^2,3^ These factors are expressed as the critical packing parameter (*C*PP = *v*/*al*), where *v* is the volume of the tail, *a* is the head group area, and *l* is the tail length of the amphiphilic molecule. As a guideline, amphiphiles with a CPP below 1/3 self-assemble into spherical micelles, between 1/3 and 1/2 into cylindrical micelles, and above 1/2 into vesicles or flexible bilayers.^3^

**Figure 1:**
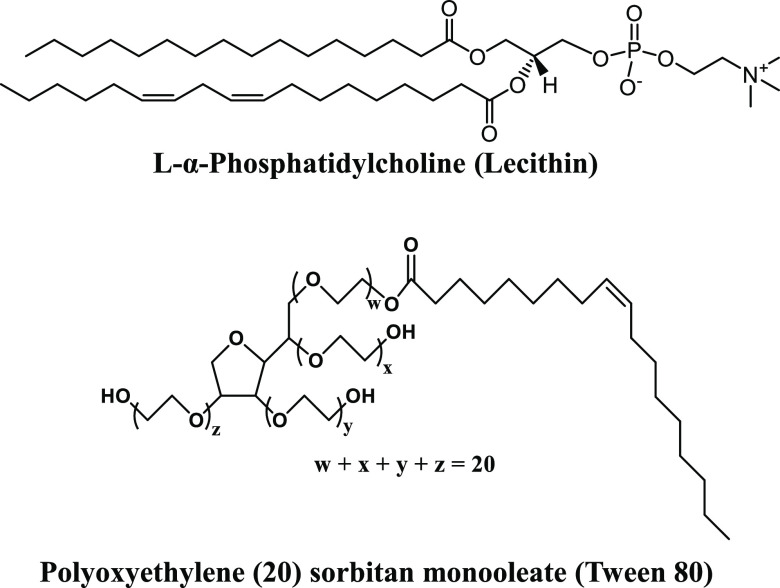
Chemical structure of l-α-phosphatidylcholine (lecithin) and polyoxyethylene (20) sorbitan monooleate (Tween 80). Lecithin is a mixture of phosphatidylcholines of varying hydrocarbon chain lengths, and the structure shown above is representative of the predominant lipid species in lecithin.

This work focuses on the aqueous self-assembly of mixtures of a zwitterionic phospholipid, l-α-phosphatidylcholine, which is commonly referred to as lecithin (L), and a nonionic surfactant, polyoxyethylene (20) sorbitan monooleate (Tween 80 or T) (Figure 1). Lecithin is a mixture of phosphatidylcholines of varying hydrocarbon chain lengths (CPP = 0.5 to 1),^4^ which favors the formation of lipid bilayers (see Section S1 of the Supporting Information for details on the lipid composition of lecithin). In contrast, Tween 80 has a CPP of 0.07^5^ and assembles into spherical micelles. Therefore, mixtures of lecithin and Tween 80 (the LT system) will likely form mixed lipid–surfactant aggregates that accommodate the geometric constraints imposed by the significant difference in their CPPs. In addition to relevance in biomedical applications as drug delivery vehicles,^6−8^ the combination of the two amphiphiles has shown significant potential as an effective dispersant for the remediation of oil spills.^9−11^ Both lecithin and Tween 80 are food-grade, degradable materials, and their use in dispersants is appealing because current dispersants contain synthetic surfactants such as the anionic dioctyl sodium sulfosuccinate that may persist in the environment. A specific finding of note is that emulsion droplets formed in the LT system are more resistant to recoalescence when compared to the traditionally used dispersant, Corexit 9500.^9^ Because mixtures of lecithin and Tween 80 (LT system) have been used in various contexts,^6−11^ there is a need to clearly elucidate the morphology of self-assembled LT nanostructures.

Recent studies have used cryogenic transmission electron microscopy (cryo-TEM) coupled with small-angle X-ray scattering to characterize the morphology of nanostructures formed during structural transitions in amphiphilic mixtures.^12−14^ The combination of these nanoscale characterization techniques was used to elegantly describe a transition from lipid vesicles to flattened sponge-like structures of bilayers containing pores.^12^ Additionally, numerous studies have reported vesicle to micelle structural transitions in mixed amphiphilic systems.^15−23^ For example, lipid/bile salt mixtures, which have been extensively investigated due to their physiological significance have shown vesicle-to-micelle transitions.^15−20^ These studies on lipid/bile salt mixtures show that the addition of bile salts such as sodium cholate to phospholipid vesicles leads to transitions from vesicles (with phospholipid rich systems) to micelles (in bile salt rich systems).^15−20^ In another system composed of lecithin/octaethylene glycol *n*-dodecyl monoether, it was reported that the addition of octaethylene glycol *n*-dodecyl monoether to small lecithin unilamellar vesicles resulted in a vesicle to micelle transition, with thread-like structures as intermediates between the vesicles and spherical micelles.^22^ Our work with the LT system adds to the class of such structures and represents a system with easily available amphiphiles.^24^ Herein, we use cryo-TEM micrograph analysis (including tilt cryo-TEM), dynamic light scattering (DLS), and small-angle neutron scattering (SANS) experiments to detailly characterize the structures formed in LT aqueous mixtures, with emphasis on elucidating the morphology of structures formed at intermediate L/T compositions.

The LT amphiphilic mixtures are prepared by first dissolving lecithin and Tween 80 in an organic solvent (such as ethanol). This is the simplest way to deliver the two amphiphiles as a dispersant because lecithin is insoluble in water, while Tween 80 is fully soluble. In a relevant and important earlier study from Bothun’s laboratory,^11^ it was shown that a concentrated mixture of lecithin and Tween 80 in water could be directly used as a dispersant. In this mixture, lecithin and Tween 80 self-assembled into colloidally stable nanostructures. While the sparingly water-soluble lecithin forms vesicles upon hydration from a dry state, and the fully water-soluble Tween 80 forms spherical micelles, the authors showed that LT mixtures when sonicated in water formed discoid structures or bicelles.^11^ Such bicelle formation by LT was earlier noted through the pioneering work by Aramaki.^24^ In the context of oil-spill dispersion, surface oil often takes the form of long streaks due to Langmuir circulation patterns^25^ on the ocean surface through wind and wave-generated counter-rotating vortices. Aerial dispersant spraying, therefore, leads to a significant amount of the dispersant contacting water rather than the solvent. An understanding of amphiphile self-assembly as the solvent dilutes away is important in understanding and optimizing dispersion efficiency.

## Materials and Methods

### Materials

l-α-Phosphatidylcholine (Soy) (catalogue number: 441601) was purchased from Avanti Polar. Polyoxyethylene (20) sorbitan monooleate (Tween 80), ethanol (reagent grade), and ethylene glycol were purchased from Sigma-Aldrich. Deuterium oxide was purchased from Cambridge Isotope Laboratories. Deionized (DI) water generated by an ELGA reverse osmosis water purification system (MEDICA 15BP) with a resistance of 18.2 MΩ·cm was used in all experiments.

### Preparation

Lecithin and Tween 80 were dissolved in ethanol at an amphiphile (lecithin + Tween 80) concentration of 9 wt % and at different lecithin to Tween 80 weight ratios. Deionized water was then added to the lecithin-tween 80 in ethanol mixture until the water concentration reached 75 wt % (lecithin and Tween 80 in water–ethanol mixture). The final amphiphilic mixtures were composed of 2.25 wt % amphiphile (lecithin + Tween 80), 22.75 wt % ethanol and 75 wt % water. An ultrasonication apparatus (Cole Parmer Bransonic Ultrasonic 8890 aquasonic 151) operated at 50 W and 20 kHz was used for sample preparation. After sonication was applied for 5 min, the samples were incubated at room temperature for at least 24 h prior to instrumental characterization.

### DLS

DLS measurements were made on a NanoBrook 90Plus PALS (Brookhaven Instruments) with 40 mW red diode laser and a 640 nm wavelength. The measurements were performed at 25 °C, and the scattering signal was collected at 90°. The autocorrelation function was measured using a logarithmic correlator and analyzed using the BIC Particle Solutions (v3.1) software to obtain the hydrodynamic size. The CONTIN algorithm was used to extract the particle size distributions from the DLS measurements.

### Small-Angle Neutron Scattering

SANS measurements were made on the VSANS Instrument at the National Institute of Standards and Technology (NIST) Center for Neutron Research (NCNR) in Gaithersburg, MD. The samples were prepared with pure deuterium oxide to generate enough scattering contrast. All measurements were conducted at 25 °C. The raw data were corrected for the empty cell and background using the macros provided by the NCNR.^26^ The data are presented as absolute intensity versus the wave vector *q* = 4π sin(θ/2)/λ, where λ is the wavelength of incident neutrons and θ is the scattering angle.

### Cryo-Transmission Electron Microscopy

Cryo-TEM imaging was done on a FEI G2 F30 Tecnai TEM operated at 200 kV. The sample was prepared using a FEI Vitrobot. Five microliters of the solution were applied to a 200-mesh lacey carbon grid (Electron Microscopy Sciences). Excess liquid was blotted by filter paper attached to the arms of the Vitrobot for 2 s to form a thin film. The sample was then vitrified by plunging into liquid ethane followed by liquid nitrogen. The vitrified sample was finally transferred onto a single tilt cryo-specimen holder for imaging. The cryo-holder was maintained below 170 °C to prevent sublimation of vitreous water. All size analyses on cryo-TEM images were carried out using ImageJ software.

## Results and Discussion

### DLS Characterization

**Figure 2:**
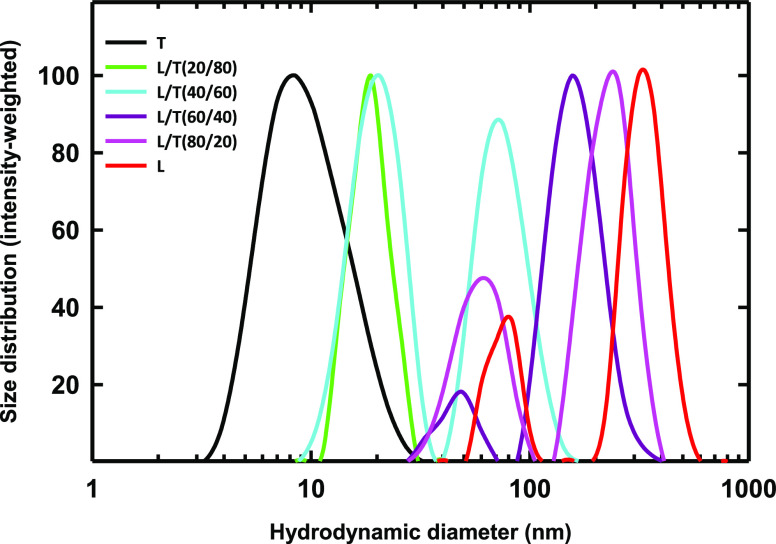
Intensity-weighted size distribution as a function of L/T weight ratio. Mixtures are prepared at an overall amphiphile (lecithin + Tween 80) concentration of 2.25 wt %.

As the first step in characterizing LT mixtures, we carried out DLS experiments at different L/T weight ratios. The intensity-weighted size distributions in Figure 2 indicate a gradual decrease in sizes present as the Tween 80 fraction is increased. In samples with lecithin alone (i.e., L/T = 100/0), two populations of nanostructures with hydrodynamic diameters (*D*_h_) of 75 nm and 338 nm are found. These nanostructures are likely to be lipid vesicles. At 60/40 L/T, smaller nanostructures with a *D*_h_ of 32 nm coexist with larger structures that are likely lipid vesicles. This change could indicate the presence of smaller vesicles or a new population of morphologically different nanostructures. The 20/80 L/T sample shows a unimodal size distribution with a *D*_h_ of 18 nm. The disappearance of larger nanostructures highlights the probable absence of lipid vesicles. Visually, the 20/80 sample is highly transparent, while the samples with a lower Tween 80 content are translucent (Figure S2). The increased optical clarity at 20/80 suggests the absence of large nanostructures that strongly scatter visible light. Tween 80 alone forms nanostructures with a hydrodynamic diameter of 9 nm, which are likely Tween 80 micelles with sizes close to values reported in the literature.^5^

### SANS Studies

**Figure 3:**
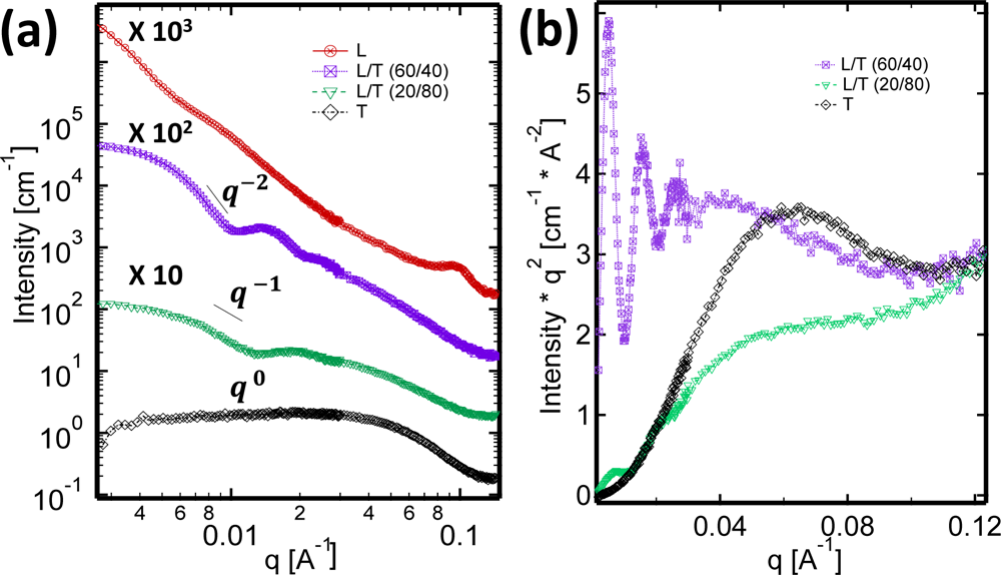
Measured SANS analysis of the LT system at different mixing ratios. (a) SANS profiles of the LT systems at different L/T ratios. The intensities scaled to allow for better viewing of the data. (b) Kratky plots of LT mixtures from the SANS data. Mixtures are prepared at an overall amphiphile (lecithin + Tween 80) concentration of 2.25 wt %.

We carried out SANS experiments on LT mixtures to probe the nanostructures’ morphology. Figure 3 shows a scaled plot of the SANS profiles (scattering intensity *I* vs scattering vector *q*) for amphiphile mixtures at different L/T weight ratios. The pure lecithin sample does not show the expected characteristic scattering signature from unilamellar vesicles (ULVs), which is a *q*^–2^ power law dependence at low and intermediate *q*.^27,28^ This sample has a small Bragg peak at *q* = 0.099 Å^–1^, which corresponds to a *d*-spacing of 63.5 Å, suggesting the presence of multilamellar vesicles (MLVs). MLV nanostructures have been reported in lecithin solutions,^29^ and our experimental *d*-spacing value agrees with the bilayer spacing of MLVs in these systems.^30,31^ Nele and co-workers have also shown that the presence of a small Bragg peak at *q* = 0.095 Å^–1^ in the SANS profiles of extruded 1-palmitoyl-2-oleoyl-*sn*-glycero-3-phosphocholine (POPC) vesicles is evidence of residual bilamellar, trilamellar, and multilamellar vesicles after extrusion.^32^ At the other extreme, the pure Tween 80 sample has a scattering profile with a plateau intensity at low *q* and a smooth curvature at high *q*, which is a characteristic of scattering from spherical micelles.^5,33^ The samples with mixed L/T content display features indicating morphological changes from vesicular to micellar structures. In contrast to the pure lecithin sample, we observe no Bragg peak at a L/T ratio of 60/40, indicating the absence of multilamellarity. The 60/40 scattering profile has a flat Guinier regime at the lowest *q*, indicating discrete objects, followed by a *q*^–2^ power law dependence, indicative of scattering from structures with a planar morphology such as lipid bilayers.^27,28^ A transition to a *q*^–1^ power law dependence at intermediate *q* appears for the 20/80 sample, indicating cylindrical structures (rod-like or wormlike micelles).^34,35^ Rod-like micelles are known to exhibit a scattering profile with a *q*^–1^ dependence at intermediate *q* with the lower and higher cutoffs of this behavior being dependent on the reciprocal of rod length and diameter.^35^ Hence, the SANS profiles suggest a transition from lipid bilayers (vesicles) to rod-like micelles to spherical micelles. We further analyzed the SANS profiles of the 60/40, 20/80, and Tween 80 only samples using Kratky plots (*Iq*^2^ vs *q*, Figure 3b).^36,37^ The Kratky plot of Tween 80 alone has a single broad peak, typical of small globular nanostructures.^37,38^ The 20/80 sample has a small peak at low *q* followed by a gradual increase in *Iq*^2^ as *q* increases. The gradual increase in *Iq*^2^ is characteristic of rods, and the small peak at low *q* is indicative of globular structures.^37^ At higher lecithin content (60/40), characteristic oscillations appear at low *q*, indicating vesicular structures.^36^ Hence, the Kratky plots suggest that rod-like aggregates (20/80) are intermediate nanostructures between vesicular structures (60/40) and small spherical micelles (Tween 80 alone).

**Figure 4:**
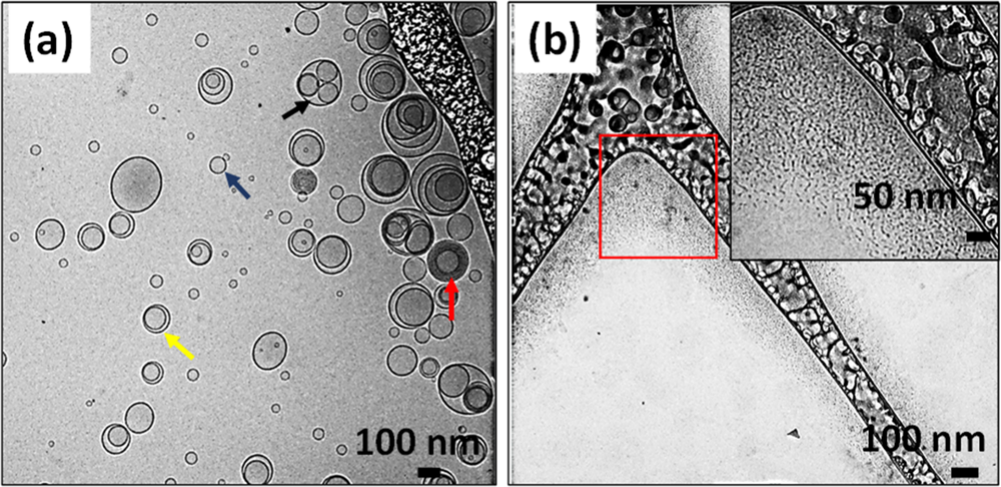
Nanostructures formed by pure lecithin and pure Tween 80. (a) Cryo-TEM of lecithin, with the blue arrow pointing to a unilamellar vesicle, yellow arrow pointing to a bilamellar vesicle, red arrow pointing to a multilamellar vesicle, and black arrow pointing to an oligo-vesicular vesicle. (b) Cryo-TEM of Tween 80 micelles. Amphiphile mixtures are prepared at an overall amphiphile (lecithin or Tween 80) concentration of 2.25 wt %.

**Figure 5:**
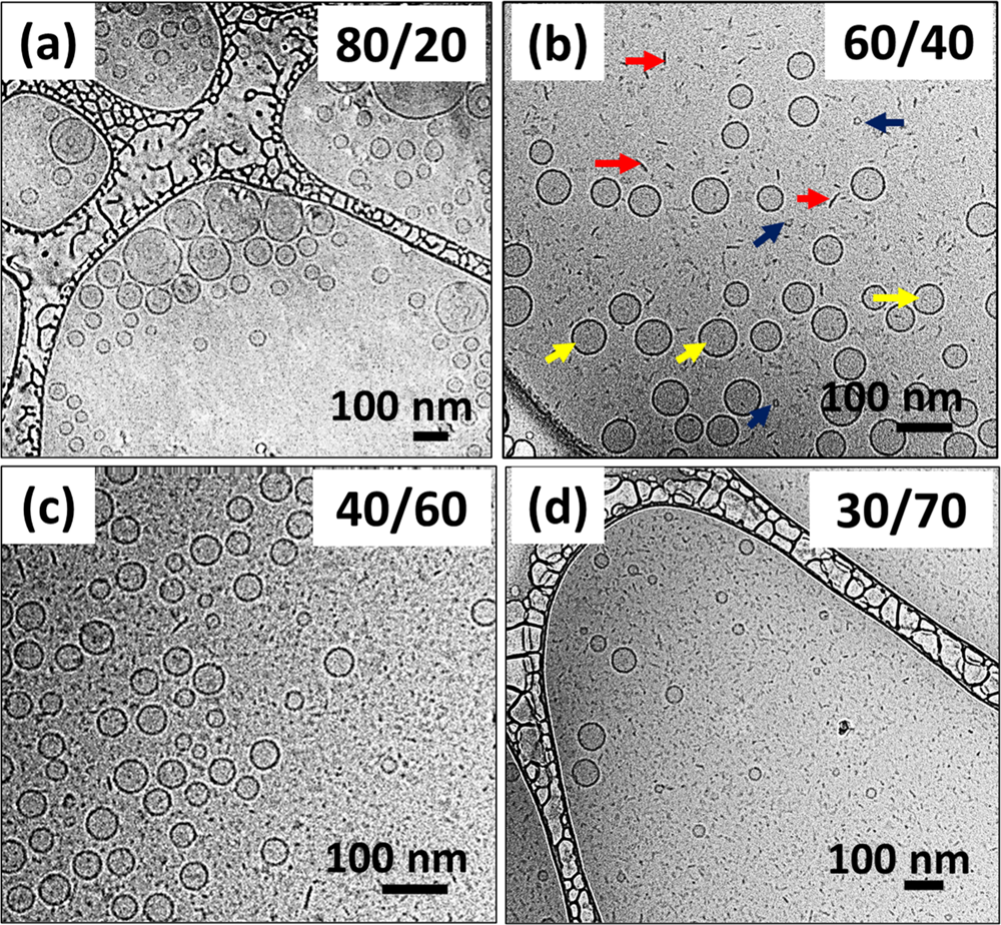
Cryo-TEM images of the samples at (a) 80/20, (b) 60/40, (c) 40/60, and (d) 30/70 L/T weight ratios. Large unilamellar vesicles are formed at 80/20. Bicelles and vesicles coexist at 60/40, 40/60, and 30/70 L/T weight ratios. The red, blue, and yellow arrows point to bicelles on an edge-on orientation and bicelles on a face-on orientation and vesicles, respectively. Mixtures are prepared at an overall amphiphile (lecithin + Tween 80) concentration of 2.25 wt %.

**Figure 6:**
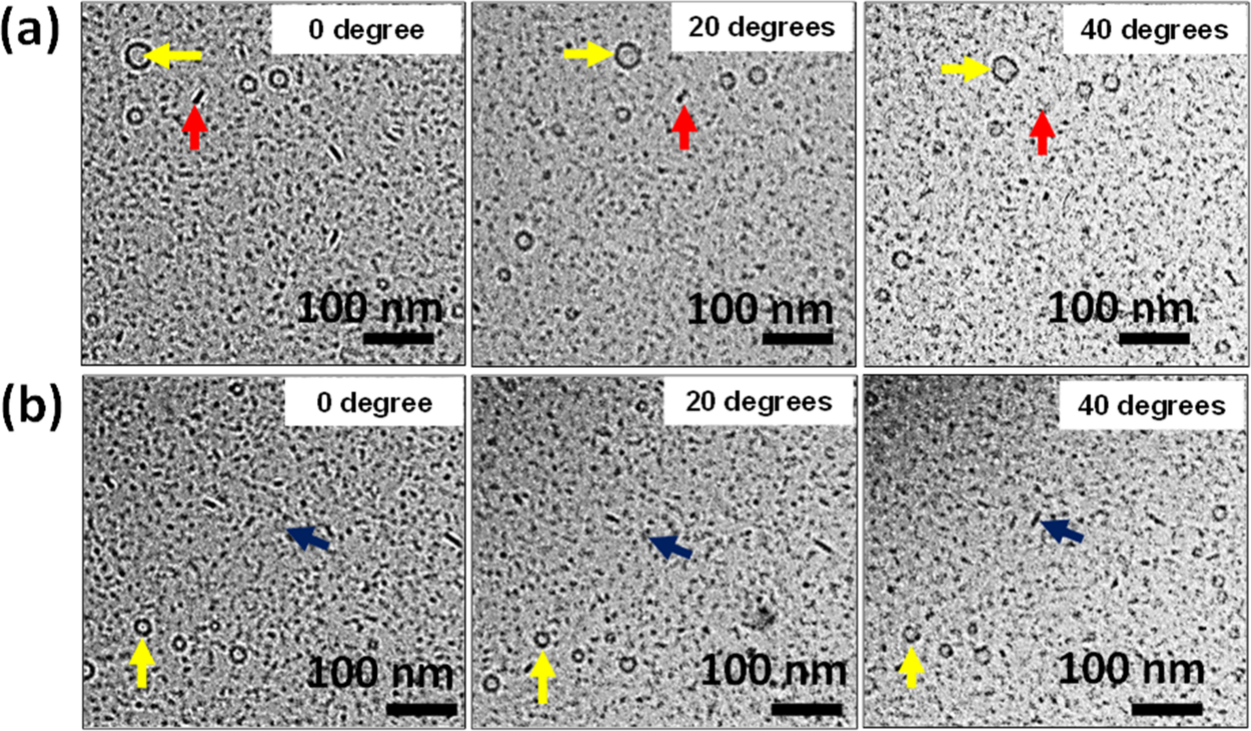
Change in nanostructures’ morphology with tilting cryo-TEM stage. (a) Change in the bicelle morphology from edge-on orientation to face-on orientation (red arrows). (b) Change in the bicelle morphology from face-on orientation to edge-on orientation (blue arrows). The vesicle morphology (yellow arrows) stays the same (spherical symmetry). LT mixture (30/70) prepared at an overall amphiphile (lecithin + Tween 80) concentration of 2.25 wt %.

**Figure 7:**
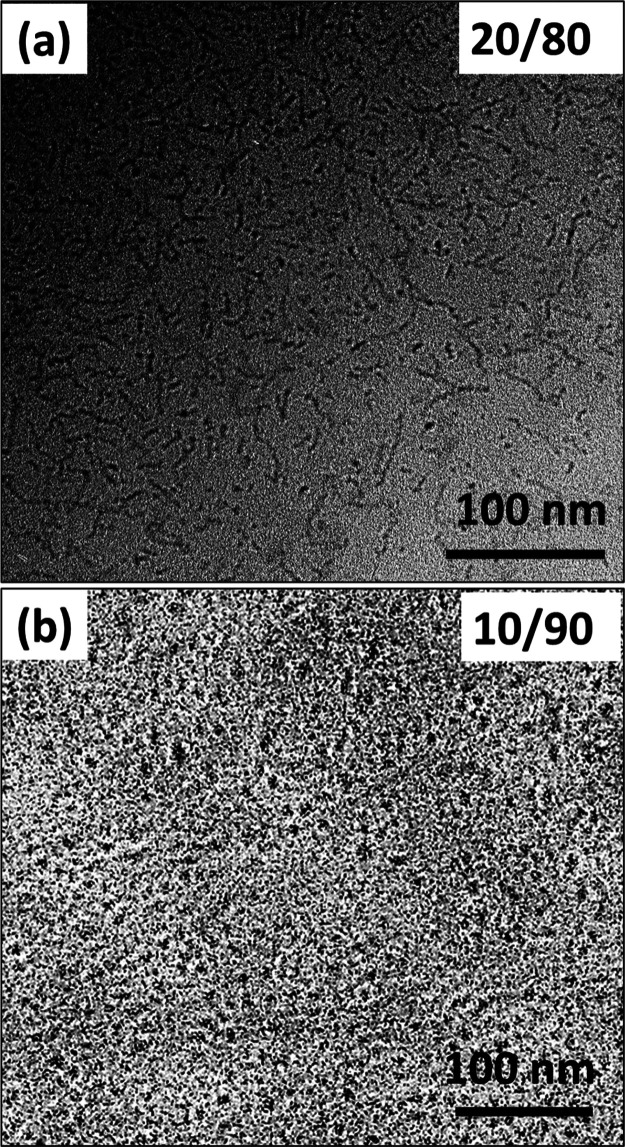
Cylindrical and spherical micelles formed at higher concentrations of Tween 80. Cryo-TEM images of the samples at (a) 20/80 and (b) 10/90 L/T ratios. Cylindrical micelles are formed at the 20/80 L/T ratio and spherical micelles are formed at the 10/90 L/T ratio. Mixtures are prepared at an overall amphiphile (lecithin + Tween 80) concentration of 2.25 wt %.

To quantify the nanostructures present, the SANS data need to be analyzed using appropriate models. We were unable to successfully model the SANS data due to the possible coexistence of different nanostructures such as lipid vesicles and disc-like and rod-like micelles. Such modeling requires knowledge of the detailed compositional and structural information of the different nanostructures as well as their relative volume fractions at every composition, which requires too many fit parameters. Nevertheless, the SANS data show clear evidence of distinct structural transformations in the samples, and our results from cryo-TEM (Figures 4–7) offer additional nanoscale understanding of the morphology of the self-assembled nanostructures.

### Cryo-TEM Imaging

We used cryo-TEM to visualize the self-assembled nanostructures present in the various samples. Pure L self-assembles into a mixture of unilamellar, bilamellar, multilamellar, and oligo-vesicular vesicles (small vesicles nested within larger bilayers) (Figure 4a). By analyzing many images of these vesicles (a total of 266 vesicles), we find that 72% of the vesicles are unilamellar, 15% are bilamellar, 10% are multilamellar, and 2.3% are oligo-vesicular. The direct observation of MLVs in the cryo-TEM images is consistent with SANS scattering features of pure lecithin (Figure 3a), where we associated the small Bragg peak at *q* = 0.099 Å^–1^ with MLVs. Tween 80 images resemble dots less than 10 nm in diameter, indicative of spherical micelles (Figure 4b).

Figure 5 shows cryo-TEM images of LT mixtures. At low levels of Tween 80, that is, at an L/T ratio of 80/20, the vesicular structures are preserved but they are now predominantly unilamellar (Figure 5a). Analysis of vesicles in this sample from many images (322 vesicles counted) reveals that only 6% of the vesicles are bilamellar, multilamellar, or oligo-vesicular. Next, at a L/T ratio of 60/40, we again see ULVs, but there is a complete absence of MLVs. The disappearance of multilamellarity at 60/40 is consistent with the SANS data (Figure 3a), where the Bragg peak was absent for this sample. In recent remarkable findings, Nele and co-workers showed that the incorporation of PEGylated phospholipids within lipid bilayers promoted the vesicles’ unilamellar character.^32^ They suggested that bulky hydrophilic components within lipid bilayers sterically hindered the lamellar stacking of lipids. Our results point to the generality of this phenomenon. Tween 80 has a large hydrophilic head group (with three oxyethylene side chains) that may sterically hinder the lamellar stacking of lecithin lipids into MLVs. As such, the increase in the vesicles’ unilamellar character may be induced by the presence of steric barriers between Tween 80-containing lecithin bilayers.

At the 60/40 L/T ratio (Figure 5b), ULVs coexist with several small structures that appear as either dark lines (red arrows) or as less visible patches (blue arrows). These dark lines and patches are a representative of disklike structures called bicelles (bilayered micelles) frozen at multiple orientations, with some that are oriented edge-on, while others are more face-on relative to the electron beam during imaging.^39^ The higher visibility (contrast) of edge-on bicelles is because there are more amphiphiles in the path of the electron beam and has been observed in earlier studies.^39−41^ It is important to note that bicelles on their edge do not appear to retain the curvature of lipid vesicles, distinguishing them from broken vesicles sometimes observed during vesicle solubilization.^42^ Image analysis reveals that the bicelles have an average diameter and polydispersity of 26 nm and 7%, respectively (40 aggregates measured). Similar disk-like assemblies have also been observed for various phospholipid mixtures as well as mixtures of phospholipids with other amphiphiles.^39,43,44^ For example, mixtures of dipalmitoyl phosphatidylcholine (DPPC) and dihexanoyl phosphatidylcholine form bicelles that effectively penetrate the stratum corneum of the skin and are used for therapeutics delivery through the skin.^43^ The coexistence of ULVs and bicelles observed from cryo-TEM for the 60/40 sample is broadly consistent with its SANS data, which indicated planar structures such as lipid bilayers. However, cryo-TEM also shows why it is hard to directly model the SANS data because such modeling requires knowing the volume fractions of both structures present.

Why do bicelles arise in LT mixtures? The reason lies in the different geometries of lecithin and Tween 80 molecules. Tween 80 has a large head, which makes it a conical structure (CPP = 0.07)^5^ and thereby it self-assembles into spherical micelles.^11^ Lecithin is composed of phosphatidylcholines of varying hydrocarbon chain lengths (CPP = 0.5 to 1)^4^ and self-assemble into lipid bilayers (vesicles).^45^ In LT aggregates, the conflicting geometric constraints imposed by lecithin and Tween 80 molecules have to be accommodated simultaneously. Tween 80 will preferentially pack at curved interfaces and lecithin at planar interfaces. Consequently, when there is sufficient Tween 80, we expect that lecithin and Tween 80 will together form bicelles in which the two sets of molecules are segregated in separate “zones”. The lecithin molecules will form the planar interface (body) of the bicelle, while the Tween 80 molecules will decorate the rims of the bicellar disk, where the curvature is high. Similar segregation has been hypothesized to occur for bicelles formed by other mixtures of amphiphiles.^44,46^ For example, Drescher and coworkers studied bicelles of DPPC and bola-amphiphiles.^44^ They showed that the DPPC molecules (CPP = 0.79) preferentially formed the bicellar body, whereas the bola-amphiphiles (CPP = 0.39) formed the rims of the bicelles.

It is also important to note that the ULVs in the 60/40 L/T sample (Figure 5b) are smaller than in the 80/20 one (Figure 5a). From image analysis (70 vesicles measured), the ULVs at 80/20 have an average diameter and polydispersity of 107 nm and 43%, respectively. In contrast, the ULVs at 60/40 have an average diameter and polydispersity of 61 nm and 5%, respectively (again, 70 vesicles were measured). Thus, as Tween 80 is increasingly inserted into the bilayers, the vesicle size decreases. This makes sense because the insertion of Tween 80 will increase the spontaneous curvature of the bilayer, and to accommodate the extra curvature, the vesicles will have to become smaller.

Next, we examine closely the cryo-TEM images for the 40/60 and 30/70 L/T samples (Figures 5c,d). As the level of Tween 80 in the LT mixture is further increased, more of the ULVs will be converted into bicelles. In the 40/60 L/T sample (Figure 5c), bicelles with an average diameter of 19 nm and a polydispersity of 20% (44 aggregates measured) coexist with ULVs with an average diameter of 53 nm and a polydispersity of 6% (70 vesicles measured). Upon further increase of Tween 80 to 30/70 (Figure 5d), there are hardly any ULVs over the TEM field of view. This is because of the reduced availability of lecithin molecules, which favor vesicles and the increase in Tween 80 molecules available for the stabilization of the bicelles’ rim. There is also a reduction in the bicelles’ average diameter (13 nm, 12% polydispersity) at the 30/70 composition (40 aggregates measured). The decrease in the bicelle size is consistent with our hypothesis that Tween 80 and lecithin are segregated at the rims and body of the bicelles. Thus, when there are fewer lecithin and more Tween 80, there are fewer molecules to form the body and more to form the rims.

To further confirm the structure in samples where vesicles coexist with bicelles, we performed cryo-TEM stage tilting. That is, we tilted the cryo-TEM stage while directly monitoring the structures present—any changes would be due to variations in the molecules in the path of the electron beam. Cryo-TEM stage tilting was performed at the 30/70 L/T composition, where bicelles are in coexistence with a few small vesicles (Figure 6). We tracked the morphology of a bicelle on an edge-on orientation, a bicelle on a face-on orientation, and a vesicle as we tilted the cryo-TEM stage. As the stage is tilted from 0 to 40°, the very visible dash in the plane of the 2D image (red arrow) changes to a less visible patch (Figure 6a), and the less visible patch (blue arrow) changes to a visible dashed line (Figure 6b). This implies that the bicelles change from an edge-on to a face-on orientation and from a face-on to an edge-on orientation as the cryo-TEM stage is tilted. Additionally, there is no change in the circular structures corresponding to lipid vesicles as the stage is tilted (yellow arrows). This confirms that these unchanged nanostructures are spherical vesicles which have a spherical symmetry.

As the amount of Tween 80 is further increased (25/75 L/T), we observe no vesicular nanostructures (Figure S3). At this ratio, small bicelles are the predominant structures formed. However, a few short thread-like nanostructures, presumably cylindrical micelles, coexist with the bicelles. With a further increase of Tween 80 to 20/80, cylindrical micelles appear to be the prevalent nanostructures (Figure 7a). These thread-like aggregates appear to coexist with circular dots. The circular shapes are less than 10 nm in size and the small thread-like nanostructures are 11–22 nm in length. A close examination of the images reveals that the short thread-like aggregates are thinner than lipid vesicle walls and have distorted edges, suggesting that they are not pieces of lamellar phase. The threads also appear curved in the plane of the 2D image, highlighting their flexible nature (i.e., low persistence length). It should be noted that the circular dots typically have a higher contrast than the flexible aggregates, perhaps indicative of a coiled globular structure or rods vitrified with their axes parallel to the beam.

In the 20/80 sample, SANS and cryo-TEM both reveal the presence of short, flexible cylinders. The emergence of cylindrical aggregates at this high fraction of Tween 80 is also consistent with packing constraints. Finally, at 10/90 L/T (Figure 7b), uniform spherical dots less than 10 nm in size are found, representative of spherical micelles. This micellar population is indistinguishable by cryo-TEM from the pure Tween 80 micelles observed in Figure 4b. This micellar phase marks the end of the structural pathway from the vesicular to the micellar phase in LT aqueous mixtures.

Given that the amphiphilic mixtures are prepared by first dissolving lecithin and Tween 80 in ethanol, the effect of ethanol on the self-assembled nanostructures was checked. To verify the effect of ethanol on the self-assembled structures, an LT mixture at the vesicle-bicelle coexistence phase (60/40 L/T ratio) was further diluted 10 times and characterized by cryo TEM (Figure S4). As shown in Figure S4, vesicles and bicelles are observed in the diluted sample, which is consistent with the observations made without further dilution (Figure 5b) and indicates that the small ethanol content does not visibly affect the self-assembled nanostructures formed. However, it is important to note that the presence of ethanol intercalated within phospholipid bilayers can enhance lipid bilayer fluidity.^47,48^ In the relevant work by Patra and co-workers, it was shown that ethanol can intercalate within lipid bilayers and form hydrogen bonds with phospholipids’ head groups, resulting in the decrease in the order of the lipid hydrocarbon chains.^48^ The increased disorganization of the hydrophobic interactions between the phospholipid hydrocarbon chains (phospholipid tails) enhances the lipid bilayer fluidity.^48^

**Figure 8:**
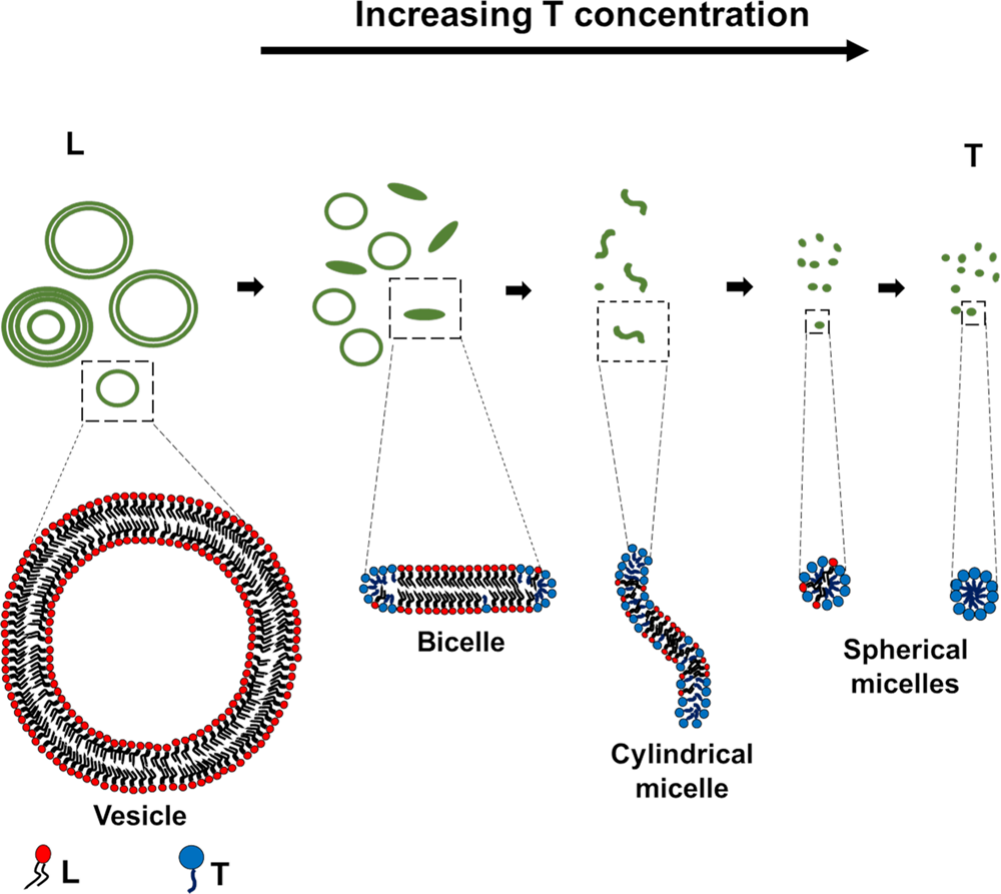
Schematic representation of the vesicle to spherical micelle structural transition in LT mixtures. The presence and morphology of the intermediate nanostructures are attributed to the balance between the favored packing geometries of lecithin and Tween 80. Even though the overall trend presented here is valid, intermediate regions between the vesicles of the pure lecithin phase and spherical micelles of the pure Tween 80 phase will show coexistence regions, where bicelles and/or rod-like micelles exist with vesicles and/or spherical micelles.

Based on the SANS results and cryo-TEM observations, we propose a vesicle-to-spherical micelle structural pathway as shown in Figure 8. First, it is recognized that the incorporation of small amounts of Tween 80 molecules within lecithin bilayers results in the reduction in multilamellarity because the bulky hydrophilic components of Tween 80 molecules within the lecithin lipid bilayers sterically hinder lamellar stacking of the lipids. In the presence of higher amounts of Tween 80 molecules, lecithin and Tween 80 molecules together form bicelles in which the two amphiphiles are segregated into regions that accommodate their preferred packing geometries. With further increase in the Tween 80 concentration, flexible worm-like micelles are formed. The emergence of these cylindrical aggregates is due to the increased packing constrain in the LT mixed aggregates because of the saturation of these nanostructures with Tween 80 molecules. Finally, the presence of extremely high Tween 80 concentrations results in the formation of spherical mixed micelles. It is important to note that Figure 8 may not fully describe the heterogeneous composition of the structures present in LT systems. Nevertheless, there are clear trends in the self-assembly as the L/T composition varies. While the overall trend presented in Figure 8 is valid, intermediate regions between the vesicles of the pure lecithin phase and spherical micelles of the pure Tween 80 phase will show coexistence regions where bicelles and/or rod-like micelles exist with vesicles and/or spherical micelles.

An important consideration in the use of LT mixtures is the choice of solvent used in dissolving the amphiphiles and as it may be expected, technologies that use these mixtures have used many different solvents based on their desired application.^24,49^ For example, Fernandes and coworkers tested the impact of solvents used to dissolve LT on the crude oil dispersion of these mixed amphiphiles.^49^ Hence, we tested the impact of the solvent used on the morphology of the self-assembled nanostructures. The nanostructures formed by using ethylene glycol and propylene glycol as a solvent were investigated. At the 60/40 L/T ratio, bicelles and vesicles are formed in the amphiphilic mixtures in the three solvents tested (Figure S5). This is consistent with the observations made using ethanol (Figure 5) and indicates that multiple solvents can be used to dissolve the amphiphiles prior to mixing in water to consistently form the self-assembled nanostructures in accordance with the phase behavior of these amphiphile mixtures in water.

## Conclusions

We have elucidated the structural transitions in LT mixtures. A detailed nanoscale understanding of the structures present as the system transitions from a vesicular to a micellar state has been established. Our cryo-TEM results reveal structural details that presents a vesicle-to-spherical micelle structural transition. The removal of vesicle multilamellarity through the incorporation of high curvature amphiphiles is a notable observation extending earlier findings^32^ and pointing to the generality of including a high curvature inducing amphiphile with a large headgroup. Emphasis has been placed on the presence and morphology of intermediate nanostructures not previously established in LT mixtures (bicelles and short, flexible cylindrical micelles). These structural transitions are due to the amphiphiles’ curvature-driven segregation of lecithin and Tween 80 molecules. For example, the planar interfaces of bicelles are predominantly made up of the long double tailed molecules of lecithin, which favor minimal curvature, while the single tailed molecules of Tween 80 primarily occupy the rim of the discoid structure. Also, the emergence of flexible rod like micelles is due to the accumulation of curvature inducing Tween 80 at the edges of sheets primarily composed of lecithin. It is important to note that the results obtained from SANS analysis were comparable to the detailed structural observations made from the cryo-TEM micrographs, highlighting the complementary use of these two techniques to elucidate the microstructure of the LT assemblies.

The translation of these fundamental concepts could advance the use of these mixtures in various applications such as membrane mimetics, drug delivery and oil spill dispersion. In the context of drug delivery, a study from the Raghavan laboratory^6^ reported that LT mixtures at the coexistence phase (vesicles and bicelles) can effectively penetrate the protective barrier of the skin and, hence, prove useful for the needle-free delivery of therapeutics into the skin. It was proposed that the presence of bicelles in coexistence with vesicles resulted in the enhanced penetration of the nanostructures into the skin because of the rapid dynamic exchange of amphiphiles between nanostructures and/or the lipid components of the stratum corneum. Additionally, work from the Nieh laboratory^50^ has shown that the morphology of self-assembled lipid-based nanoparticles affects their uptake by cancer cells. It was reported that a cellular uptake of disk-like amphiphilic aggregates is significantly higher than that of vesicles based on studies with different human cancer cell lines (CCRFCEM, KB, and OVCAR-8).^50^ The authors postulated that the higher cellular uptake observed in the disk-like structures was because the disk-like morphology enables a larger surface contact area to interact with the cell membrane.^50^ These earlier studies indicate that an added understanding and thorough characterization of the nanostructures formed by LT mixtures are useful in technologies related to drug delivery.

## Supporting Information Available

Lipid composition of l-α-phosphatidylcholine (soy); appearance of LT mixtures at different L/T ratios; cryo-TEM of an LT sample at a 25/75 L/T ratio; cryo-TEM of the nanostructures formed in a diluted LT mixture; and cryo-TEM of the nanostructures formed by dissolving the amphiphiles in different solvents (PDF)
