# Supplementary material for: Transformation of Lipid Vesicles into Micelles by Adding Nonionic Surfactants: Elucidating the Structural Pathway and the Intermediate Structures
Source: J Phys Chem B. 2022 Mar 14;126(11):2208–16. doi: 10.1021/acs.jpcb.1c09685 (PMC8958590; doi:10.1021/acs.jpcb.1c09685)
Supplement: Supplementary file 1 — jp1c09685_si_001.pdf [file jp1c09685_si_001.pdf]

Supporting Information

Transformation of Lipid Vesicles into Micelles by Adding Nonionic Surfactants: Elucidating the Structural Pathway and the Intermediate Structures

Igor Kevin Mkam Tsengam[1], Marzhana Omarova[1], Elizabeth G. Kelley[2], Alon McCormick[3], Geoffrey D. Bothun[4], Srinivasa R. Raghavan[5], Vijay T. John[1]*

[1]Department of Chemical and Biomolecular Engineering, Tulane University, 300 Lindy Boggs Building, New Orleans, LA 70118, USA

[2]Center for Neutron Research, National Institute of Standards and Technology, Gaithersburg, MD, 20899, USA

[3]Department of Chemical Engineering and Material Science, University of Minnesota, 421 Washington avenue SE, Minneapolis, MN 55455, USA

[4]Department of Chemical Engineering, University of Rhode Island, 51 Lower College Road, Kingston, RI 02881, USA

[5]Department of Chemical and Biomolecular Engineering, University of Maryland, College Park, MD 20742, USA

* Corresponding author: Phone: 504-865-5883. E-mail: vj@tulane.edu

S1: Lipid composition of L-α-phosphatidylcholine (Soy)

L-α-phosphatidylcholine (Soy), purchased from Avanti (catalog number 441601) was used in this work. This is a phospholipid composed of 97.2 wt % phosphatidylcholine and 2.8 wt % lysophosphatidylcholine. The fatty acid composition is presented in the table below (fatty acids are listed in their short notation, with the first digit indicating the number of carbon atoms and the second digit the numbers of cis-double bonds).

Table S1: Fatty acid composition of L-α-phosphatidylcholine (Soy)

**Fatty acid**	**Wt %**
16:0	14.9
18:0	3.7
18:1	11.4
18:2	63.0
18:3	5.7
Unknown	1.2

S2: Appearance of LT mixtures at different L/T ratios

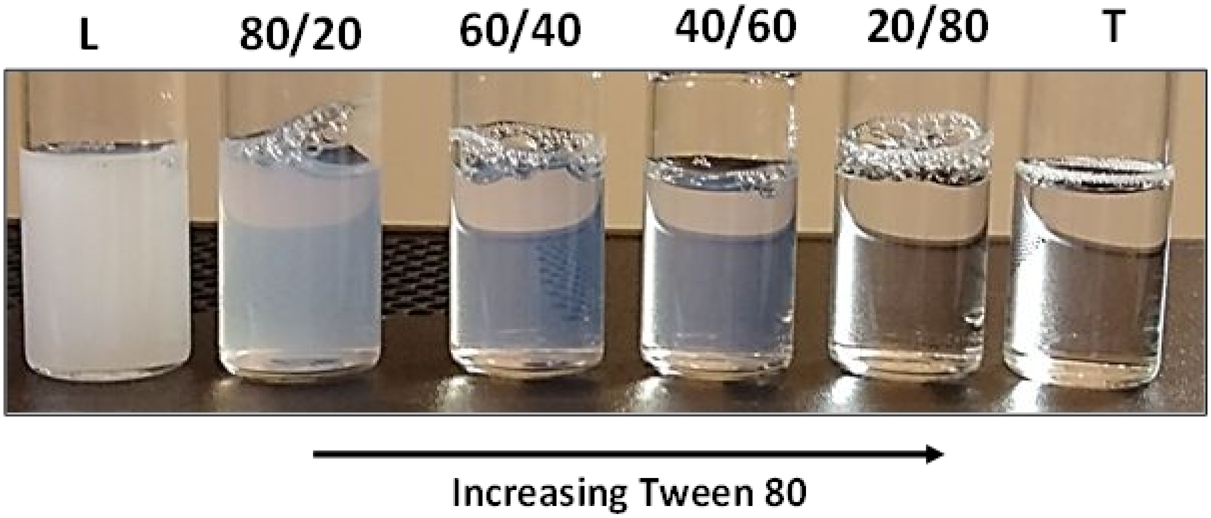

Figure S2: Appearance of LT mixtures at different L/T ratios. The observed turbidity changes are caused by changes in composition.

S3: LT mixture at 25/75 L/T ratio

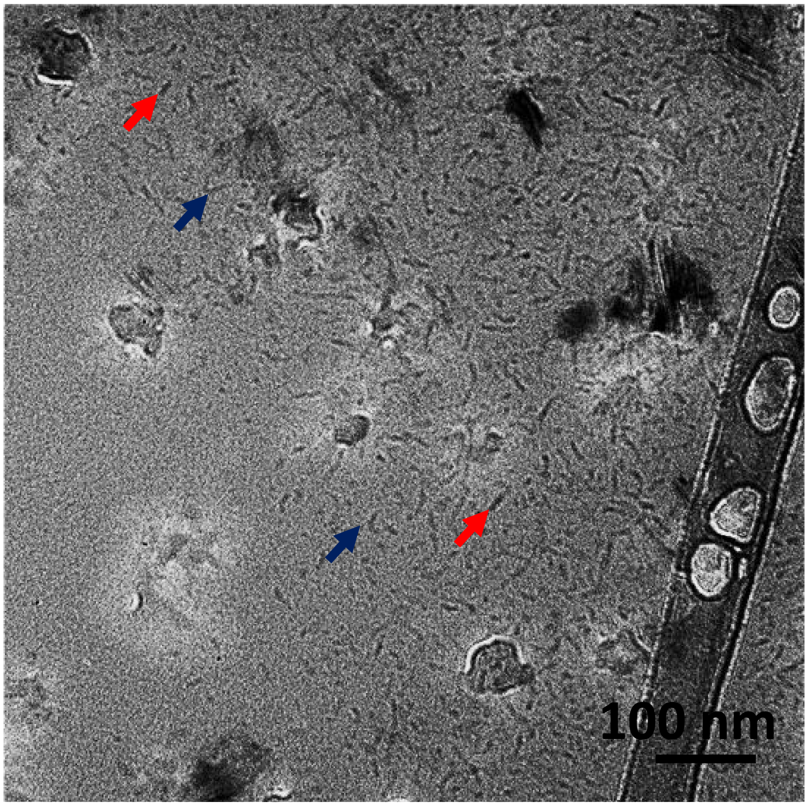

Figure S3: Cryo-TEM of an LT sample at 25/75 L/T ratio. Small bicelles and short thread-like structures are the predominant structures formed. Bicelles (red arrows) and short thread-like structures (blue arrows).

S4: Nanostructures formed in a diluted LT mixture

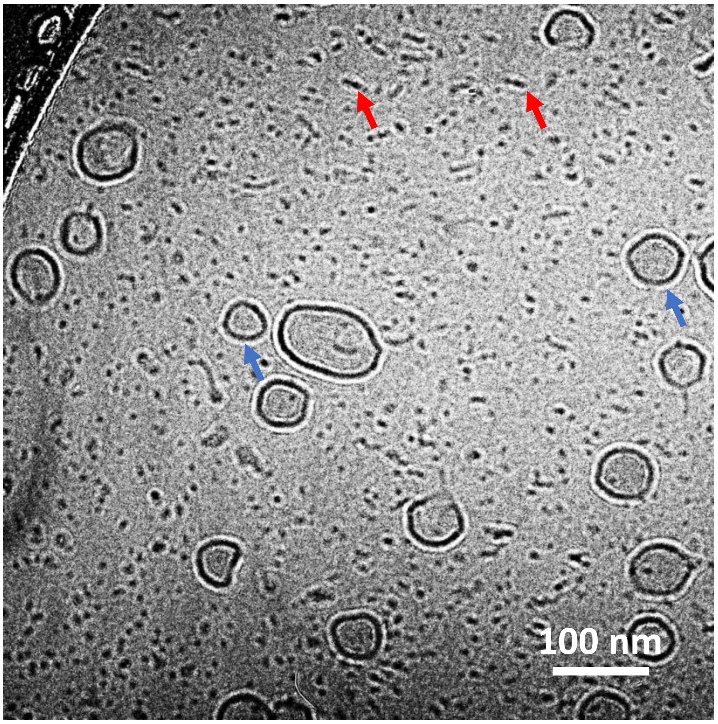

Figure S4: Cryo-TEM of the nanostructures formed in a 10 times diluted LT sample. L/T mixture at a 60/40 ratio. Bicelles and vesicles coexist at 60/40 weight ratios. Bicelles (red arrows) and vesicles (blue arrows).

S5: Nanostructures formed by dissolving the amphiphiles in different solvents

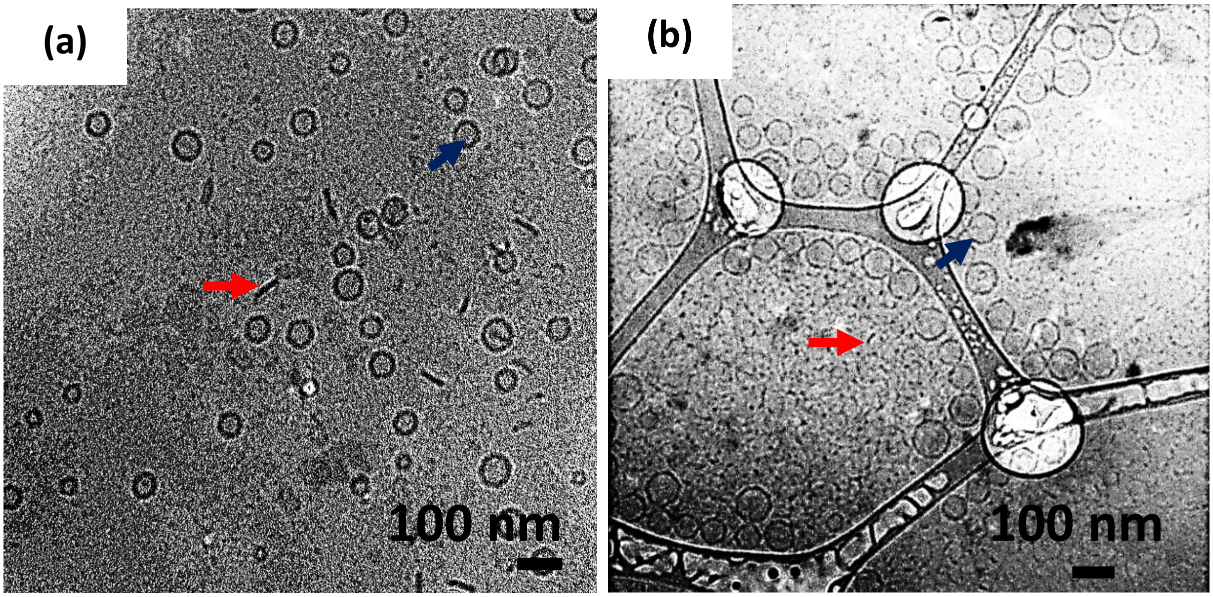

Figure S5: Cryo-TEM of the nanostructures formed by dissolving the amphiphiles in different solvents. (a) ethylene glycol and (b) propylene glycol. L/T mixture at a 60/40 ratio. Bicelles (red arrows) and vesicles (blue arrows) are formed in the amphiphilic mixtures in the different solvents tested.
